# “What does this mean for our future?” uncertainty management in mothers’ narratives about the diagnosis and birth of their child with Down syndrome

**DOI:** 10.1371/journal.pone.0313195

**Published:** 2024-11-06

**Authors:** Xavier Scruggs, Shannon Dier, Caleb A. Schlaupitz, Katherine A. Karayianis, Angela F. Lukowski, Jennifer G. Bohanek

**Affiliations:** 1 Department of Communication, University of Missouri, Columbia, Missouri, United States of America; 2 Department of Human Development and Family Science, University of Missouri, Columbia, Missouri, United States of America; 3 Department of Psychological Science, University of California, Irvine, Irvine, California, United States of America; 4 Department of Psychological Sciences, University of Missouri, Columbia, Missouri, United States of America; American University of Beirut Medical Center, LEBANON

## Abstract

Pregnancy and childbirth are uncertain experiences that become even more so when parents receive an unexpected medical diagnosis for their child. In the present study, we document sources of uncertainty and the tools used to manage uncertainty in 44 mothers’ narratives about the birth and diagnosis of their child with Down syndrome (DS); we also explore variability in the sources of uncertainty and uncertainty management tools as a function of whether mothers received a prenatal or postnatal diagnosis of DS for their child. Across our sample, thematic analysis revealed four sources of uncertainty in mothers’ narratives: navigating dissonance between parents and providers during diagnosis, managing disclosure of the diagnosis to others, anticipating child-centered challenges and adjusting developmental expectations, and anticipating family-centered challenges and adjusting familial expectations. Analyses also revealed four ways that uncertainty was managed: finding balance between parents and providers during diagnosis, reducing knowledge gaps by seeking information, pursuing support and building positive interpersonal relationships, and pursuing support and building positive relationships in DS communities. These findings, along with potential nuance based on whether mothers received a prenatal or postnatal diagnosis of DS for their child, have important implications for healthcare providers and medical professionals regarding how to best communicate DS diagnoses to families as a means of understanding and ultimately reducing the uncertainty they experience.

## Introduction

Down syndrome (DS) is the most common chromosomal abnormality worldwide [1]. Resulting from an extra copy of the 21st chromosome in some or all of an individual’s cells, DS is often associated with distinctive facial features, physical health issues (e.g., heart problems, low muscle tone, thyroid issues), and mild to moderate intellectual disability, though a great deal of individual variation exists [2]. Although DS can be diagnosed prenatally through maternal blood draw with high accuracy [3], most mothers learn of their child’s diagnosis after birth [4, 5]. Regardless of when the diagnosis is provided, families manage significant uncertainty as they navigate their new reality and the immediate and long-term variability in physical, cognitive, and social outcomes for individuals with DS [6, 7]. The present study was conducted to qualitatively examine (1) sources of uncertainty and (2) uncertainty management tools in mothers’ narratives about the birth and diagnosis of their child with DS. We also explored (3) whether sources of uncertainty and management tools differed as a function of whether mothers received a prenatal or postnatal diagnosis of DS for their child.

One potential source of uncertainty for mothers is how the diagnosis is delivered. The provision of DS diagnoses varies widely with regard to the timing of the diagnosis (i.e., prenatally or postnatally), where parents learn this information, and the specific details about DS that are provided [5–7]. Parents often report that medical professionals focus primarily on the negative features of DS when providing a diagnosis [8] and that some of the provided information is inaccurate (e.g., that individuals with DS never live independently or hold gainful employment [5]). Regardless of when the diagnosis is provided, parents initially report experiencing complicated emotions including shock, worry, grief, anger, fear, and helplessness [8–10]. Research indicates that mothers vividly remember the details of their child’s DS diagnosis decades after the event occurred and that positive support from medical professionals at the time of diagnosis may reduce the intensity with which these memories are recalled [11]. Examining how parents manage uncertainty after learning about their child’s DS diagnosis may also help clarify how medical professionals can better support patients and families during this time.

Previous research has primarily focused on how parents adjust to a DS diagnosis over time, often through the lens of stress and coping [12, 13], family stress [14, 15], or post-traumatic growth [16]; fewer studies have focused on how parents navigate the experience of receiving the DS diagnosis. Researchers [8] have described processes of adjusting to the DS diagnosis and identifying support resources as unifying themes across the experiences of parents receiving a prenatal or postnatal diagnosis. The role of prenatal screening and conversations with medical professionals were also highlighted in a recent sample of parents who received postnatal diagnoses [10]. Utilizing grounded theory, previous researchers [9] indicated that caregivers engage in sense-making processes after learning of the child’s DS diagnosis with a focus on rescuing hope for the future. Taken together, these studies suggest that information gathering and support seeking are key components of how parents manage the unknowns associated with their child’s DS diagnosis [8–10].

One framework for understanding how parents adjust to a diagnosis of DS is uncertainty management theory. According to this perspective, uncertainty occurs when information is “unavailable or inconsistent” and situations are “ambiguous, complex, unpredictable, or probabilistic” (p. 478); as a result, individuals feel insecure about whether they have the knowledge necessary to navigate the situation [17]. This framework is commonly used to identify sources of uncertainty as well as the tools used to manage that uncertainty. Previous studies have examined how families manage uncertainty in a variety of health contexts, including type 1 diabetes [18], vascular anomalies [19], and neonatal intensive care [20]. Uncertainty management provides a framework broad enough to enable identification of unique sources of uncertainty and management tools for different health conditions while also allowing for flexibility in determining to what extent the family or other individuals are involved in these processes.

The primary purpose of the present analysis was to examine sources of uncertainty and uncertainty management tools in mothers’ narratives about the birth and diagnosis of their child with DS. As an additional exploratory step, we examined whether sources of uncertainty and uncertainty management differed in the narratives of mothers who received a pre- or postnatal diagnosis of DS for their child. Previous research has indicated that, although maternal experiences vary in relation to the timing of diagnosis [6, 7], parents experience many of the same emotions and concerns regardless of when the diagnosis is provided. Nevertheless, parents who receive a prenatal diagnosis commonly express that the additional knowledge and time to prepare before the birth of their child was helpful [8, 9]. As such, it is possible that mothers report unique sources of uncertainty and management tools depending on the timing of diagnosis, a finding which would be helpful and informative for medical practitioners as they work towards best practices in supporting parents who have children with DS.

## Method

### Participants

Forty-six mothers of children with DS were recruited to participate; 44 mothers completed the study (two mothers were excluded due to poor interview audio quality). Demographic information is shown in Table 1. Mothers were recruited from Facebook support groups for families of children with DS. Eligible mothers were at least 18 years old, were comfortable engaging with the research team in English, and had only one biological child with DS who was 1–17 years old. At the end of the study, mothers received a $20 electronic gift card.

**Table 1:** Demographic characteristics (means ± standard errors).

	Group		
	Overall (n = 44)	Prenatal (n = 18)	Postnatal (n = 26)
Mother and family variables			
Age at childbirth (years)	35.81 ± .79	36.60 ± 1.22	35.02 ± 1.02
Number of biological children	2.59 ± .27	2.22 ± .42	2.96 ± .35
Race (% White)	93%	90%	96%
Ethnicity (% non-Hispanic)	86%	94%	81%
Marital status (% married)	96%	94%	96%
Education (% ≥ college degree)	75%	78%	73%
Income (% ≥ $75,000 per year)	81%	89%	76%
Religious services (% ever attended in past year)	59%	50%	65%
Obtained prenatal care (% yes)	100%	100%	100%
Started in first trimester (% yes)	98%	94%	100%
Participation in support groups (% yes)	98%	94%	100%
Child demographics			
Age at time of study (years)	6.40 ± .66	5.76 ± 1.02	7.04 ± .85
Sex (% female)	50%	56%	46%
Presence of siblings (% yes)	76%	61%	92%
Born 3 or more weeks early (% yes)	25%	39%	15%
Neonatal Intensive Care Unit stay (% yes)	41%	56%	31%
Participated in early intervention (% yes)	100%	100%	100%
Age at start of early intervention (months)	3.53 ± .31	3.71 ± .48	3.35 ± .38
Child functioning at the time of the study			
Physical health	5.54 ± .17	5.50 ± .26	5.58 ± .22
Cognitive functioning	4.37 ± .17	4.56 ± .25	4.19 ± .21
Independent functioning	4.02 ± .22	4.28 ± .33	3.77 ± .27
Activities of daily living	3.44 ± .21	3.89 ± .33	3.00 ± .27

**Note.** Descriptive statistics are provided above; information on statistically significant group differences may be obtained from the corresponding author. Parents rated child functioning on scales ranging from 1–7; higher scores indicate better functioning. Because of an issue with the online questionnaire used to collect the data, some participant responses (n = 4) were missing on the question associated with activities of daily living. The missing values were replaced with the middle option on the 7-point scale (4) to avoid biasing the data in either direction; these data are presented here. Although the means and standard errors differ somewhat when the raw data were analyzed, the pattern of effects remains unchanged.

### Procedure

This study was registered as self-determined exempt with the Institutional Review Board at the university at which the data were collected. The procedures reported below are part of a larger study which included additional narrative and questionnaire data; those measures are outside the scope of the present report and will be featured in future manuscripts.

Mothers were recruited and tested from April 12, 2019 to November 12, 2019. Potential participants clicked a link on the recruitment advertisement and were directed to a study information sheet (a waiver of signed informed consent). Participants answered a multiple choice question confirming their willingness to participate before clicking forward to access an online screening questionnaire; those who met the eligibility criteria were directed to a demographics questionnaire. Mothers were then directed to a website through which they scheduled a one-hour video chat interview with a research assistant (average delay from questionnaire completion to online interview = 10 days; range 1–51 days). Narratives were collected with video and audio enabled to permit naturalistic interactions between the participant and the researcher, but only the audio was recorded for later transcription and analysis.

During the video chat interview, mothers were asked to discuss their child’s birth story, the time they learned of their child’s DS diagnosis, and their child’s most recent birthday (this last event is outside of the scope of the present study, although it has been included in our previous quantitative work [21] with this sample and will be qualitatively examined in future reports). Events were discussed in chronological order (i.e., mothers who received a prenatal diagnosis of DS for their child reported on their child’s diagnosis first, whereas mothers who received their child’s DS diagnosis after birth discussed their child’s birth story first). Because we recognized that discussing the diagnosis and birth events in particular could be challenging for participants, we attempted to minimize any distress they might experience in multiple ways: (a) The participants read an online waiver of written informed consent (a study information sheet) before engaging in any study procedures; this document indicated that participants could skip any question they did not want to answer and that they could discontinue their involvement in the study at any time. (b) We attempted to minimize participant distress and potential rumination about the events discussed in the interview by asking all mothers to discuss their child’s most recent birthday–an event that was presumed to be positive–as the last event. (c) Research assistants were trained to be active listeners during the interview (e.g., by nodding in agreement), but were instructed not to probe for additional information or ask follow-up questions so as to avoid focusing on aspects of the events that may have been distressing to the participants. In these ways, mothers were allowed to talk about whatever aspects of their experiences they wished to discuss, and they were in charge of narrating their own experiences as they preferred.

Mothers were encouraged to take as long as necessary to discuss each event and to share as many details as possible, including negative and positive emotions. The duration of the recordings was similar across events (average duration = 6 minutes; range from 1–23 minutes for the diagnosis event; range from 1–26 minutes for the birth event). The narratives were transcribed by an online service and reviewed for accuracy by two research assistants before coding.

### Narrative coding

Open coding was conducted using a flexible phronetic iterative approach [22, 23] that allowed for consideration of both existing frameworks (top-down) and emergent meaning (bottom-up) components. Drawing on established procedures for thematic coding [24], the first and fourth authors initially read the entire set of birth and diagnosis narratives to familiarize themselves with the data. Next, these authors independently reviewed the narratives from five participants and began generating codes to describe sources of uncertainty and uncertainty management tools [17]. They also noted exemplars in the data that had not yet been captured in previous research and created new coding categories to reflect these contributions. After reflecting on their initial coding, these authors met with the larger research team and began to combine codes into higher- and highest-level themes [23, 25]. A codebook was created that included definitions and exemplars from each theme and served as a “living document” (p. 122) [26] that was updated as coders read new narratives and refined and named themes.

The first and fourth authors independently coded multiple transcripts each week and then met to discuss and compare their coding. This team-based coding approach allowed for efficiency and consistency across codes as team members relived and understood the experiences of the participants through collaborative deliberation and critical reading, thereby ensuring valid coding [26, 27]. The coders reached consensus on coding when they both agreed on whether and how the specified text should be coded. If consensus was not reached, the last author moderated the discussion and assisted in final coding decisions. After each meeting, the codes for each narrative were reviewed by the last author; all questions and discrepancies were discussed and resolved. Once all of the narratives were coded, the first and last authors met to review and solidify themes and subthemes that were identified as sources of uncertainty and, separately, management tools. They read through each coded segment and redefined subthemes and higher-level themes as needed so as to accurately represent the data. All coding and data management was completed in MAXQDA 2022 [28].

## Results

The results are presented in three main sections: (1) themes reflecting mothers’ sources of uncertainty; (2) themes that describe the tools mothers used to manage uncertainty; (3) an exploratory examination of whether sources of uncertainty and management tools may have varied as a function of the timing of DS diagnoses. Pseudonyms are used in the transcript excerpts shown below to protect the identities of the participants. Disfluencies and filler words (e.g., “you know,” “like”) have been removed from excerpts for clarity in reading.

### Sources of uncertainty

Four primary themes captured the sources of uncertainty in mothers’ stories: navigating dissonance between parents and providers during diagnosis, managing disclosure of the diagnosis to others, anticipating child-centered challenges and adjusting developmental expectations, and anticipating family-centered challenges and adjusting familial expectations. Subthemes within two of the thematic categories (navigating dissonance between parents and providers during diagnosis and anticipating child-centered challenges and adjusting developmental expectations) are shown in bold text the first time they are introduced.

#### Navigating dissonance between parents and providers during diagnosis

The context surrounding the delivery of their child’s DS diagnosis was the most frequent source of uncertainty for mothers. Both verbal and nonverbal communication about the DS diagnosis contributed to uncertainty, and this manifested in two ways: a **mismatch in communication or knowledge between providers and parents,** and **providers focusing on the negative aspects of DS**.

Mothers frequently expressed that providers did not deliver the diagnosis in a way that permitted them to fully comprehend the information. Rather, mothers perceived a mismatch between the type of communication they would have preferred, their level of medical understanding, and what their providers gave them. For example, Tammy explains how she learned of her son’s diagnosis in the hospital after his birth:

“I was just watching TV, and the pediatrician comes in without [my baby]. And he doesn’t even say, ‘Hey, let’s wake up your husband, because I’m getting ready to give you life-changing news.’ He just walks in. He says, ‘I’m Dr. Whatever-his-name-was.’ I don’t even remember what his name was. And he said, ‘We think [your baby] has DS.’ And it was just that blunt. And I immediately started crying, and he just keeps talking. He just starts trying to tell me what DNA is… I already know about DNA. I knew what DS meant, but to hear someone say that this is your child who has it when you had no inkling, it was just such a shock.”

Though the words the provider used to give the diagnosis may be medically sound, Tammy recalls the process by which the diagnosis was delivered as abrupt and insensitive because the doctor did not respond to her emotional reaction or ensure that her husband was available to provide emotional support. Even with some pre-existing knowledge about DS, the manner of delivery increased Tammy’s uncertainty and distress upon learning that her child had this diagnosis.

In other cases, the delivery of the diagnosis increased uncertainty when information was not provided to mothers in a timely fashion. For example, Morgan suspected her child might have DS when she was born, although it was not confirmed at the time by her medical team:

“I remember doing a double-take and being like, ‘Oh my gosh, my baby has DS.’ So, it was kind of an emotional roller coaster, because I could tell by looking at her, and I was sure that all the other medical people in the room, like the nurses and midwives and the pediatric resident, I’m sure they knew… . And I’m just freaking out, ‘Oh my gosh, is anybody going to say anything about it?’”

Within minutes of her child’s birth, Morgan grappled with uncertainty about whether her daughter had DS and repeatedly questioned whether she should verbalize her concerns to her husband and the medical team. The lack of information increased her sense of unease during this critical time. Other mothers felt they received incomplete or conflicting information from medical providers when the diagnosis was given. For example, Maya attempted to clarify the results of an amniocentesis: “I asked what type of DS, because I had been Googling like crazy, and I knew that there was a mosaic DS and translocation, and he was like, ‘What are you talking about? It’s just DS.’” Maya’s healthcare provider seemed to lack familiarity with DS and did not appear receptive to Maya’s attempts to learn more about her child’s specific condition, which increased Maya’s uncertainty about continuing the pregnancy.

When information about DS was provided in medical conversations, mothers reported that providers who focused on the negative aspects of DS in the absence of a more balanced perspective increased their uncertainty. For example, Clara explained how her provider focused on stereotypes and potential developmental limitations associated with a DS diagnosis:

“He also mentioned something like, ‘Oh, you know what, this boy is going to be very calm. He’s not going to move. He’s not going to walk maybe until he’s three years old…. And so he was giving me all this, ‘Maybe he won’t. Maybe he will not do this or this.’ And that just contributed to my fear…. I was waiting for the ‘but he’s going to do these other things,’ but that never happened…. So I was just so worried and afraid, and I didn’t know what the future would be for him.”

Clara described how this emphasis on what her child may not be able to do intensified her uncertainty and increased her desire to learn more about positive possibilities. Similarly, Hailey discussed how her physician emphasized the health concerns associated with DS:

“That’s when she mentioned all of the risks associated with having a child with DS and heart defects. She told us the worst of the worst, that she could have [a] feeding tube for her whole life, that many babies don’t even make it to birth and that if she did make it, she might not even make it to two years based on her heart defect.”

Elizabeth went on to indicate that the information the physician provided contrasted with her understanding that the needs and medical conditions of individuals with DS can vary widely. She explained that this focus on the worst possibilities increased the uncertainty she and her husband faced as they discussed whether they should terminate the pregnancy to spare their child pain and suffering. Several mothers mentioned providers’ talk about abortion in relation to emphasizing the negative aspects of DS, indicating it compounded their uncertainty when the medical team appeared to suggest termination as the optimal choice for the family.

Although there were too few instances in our data to warrant full inclusion here, a subset of mothers also mentioned negotiating structural barriers (e.g., cost of additional testing, difficulty locating specialists) and the challenges of managing uncertainty in the context of abortion restrictions. We return to these important issues in the Discussion, as they may be more prevalent in other samples or communities of mothers.

#### Managing disclosure of the diagnosis to others

After learning of their child’s DS diagnosis, mothers had to navigate the process of sharing the news with family and friends. As mothers had unique concerns and thus employed different approaches to sharing the diagnosis, no clear or consistent subthemes were identified. At times, mothers were uncertain when or how to best share the diagnosis with others, as illustrated by Miley: “Everybody was waiting for us to tell them, like ‘Hey, she’s here. She’s born.’ We took a really long time to tell family because we didn’t know how to tell family.” Having learned her daughter may have DS shortly after her birth, Miley’s explanation highlights the juxtaposition of sharing the news of both her daughter’s expected birth and her unexpected diagnosis.

Uncertainty was often related to concerns about how others would react to the child’s diagnosis. Whereas some mothers were uncertain as to how a specific loved one would react, other mothers expressed more general concerns. For instance, Jennifer recalled how people responded when another mother she knew had a child with DS: “I knew that people would be scared or nervous or overreact or not know what to say…. When her son was born, there was just this, ‘Oh, she’s had a baby, but he has DS.’ And it was very, kind of, negative.” This example suggests that mothers were aware that people may hold negative beliefs and assumptions about people with DS, contributing to their uncertainty regarding how best to manage disclosing the news to others.

Uncertainty also resulted when one parent was aware of the diagnosis before the other and was confronted with not knowing when or how to communicate the news to their partner. For example, Morgan, the mother who suspected her daughter had DS upon seeing her after birth, expressed uncertainty whether she should communicate this concern to her husband. After being moved to a postpartum room, Morgan eventually told her husband, “I’m not trying to freak you out or anything, but I’m like 99% sure that our daughter’s got DS.” She then learned that her husband was wondering the same thing. In this case, concern about potentially upsetting their partner increased uncertainty surrounding disclosure of the diagnosis.

When mothers did share the diagnosis with others, they were faced with managing others’ emotions as well as their own. Maya expressed how this ongoing process of disclosure increased her uncertainty:

“It’s really odd when you get the diagnosis prenatally because you spend a lot of time not being able to deal with your own emotions because every single person you tell you have to kind of counsel them as well. So there was a lot of that. Like telling my family and friends, coworkers, and having to tell all them, ‘No, it’s going to be OK.’”

For these mothers, the process of managing their child’s DS diagnosis was complicated by the added uncertainty of how others would react to learning the diagnosis and, at times, having to manage the other person’s emotions and uncertainty in reaction to this news.

#### Anticipating child-centered challenges and adjusting developmental expectations

Mothers also discussed the uncertainty that resulted from the potential long-term future implications of the DS diagnosis for their child’s social and physical development. This uncertainty was reflected in two subthemes: **navigating societal and personal stereotypes** that may impact social acceptance of their child and **managing health concerns** that might affect the child’s physical quality of life.

Uncertainty pertaining to social acceptance was often expressed as concern that others might not accept or would not value their child, as Nadia explained:

“I think the sadness I felt was that I was bringing her into a world that may not accept her. And I wasn’t sad I had a baby with DS. I was more sad that the world was not a better place for her.”

Nadia positions her concerns for her child’s acceptance against her perception of how society views those with DS. Even though her own feelings for her child did not change because of her condition, she worried that a lack of acceptance from others would contribute to a more difficult life for her child.

Other mothers focused more on friendships, peer relationships, and social experiences that might be difficult for their children, as indicated by Miley:

“It was never really like a financial concern that we had for her. It was more like, “Who’s going to be there to be her emotional support, to make sure that she’s taken care of, to make sure that she’s protected?”

Miley was uncertain about her child’s future relationships and whether she would be able to experience close interpersonal connections with others, including unconditional support, trust, and security.

Further, some mothers’ uncertainty was evident when reflecting on how their child’s diagnosis may impact their identity, capabilities, and goals; in other words, mothers experienced uncertainty about their long-term expectation of who their child might become. For example, Carol explained this shift in perspective:

“You have your picture, your son, the perfect son, and that was like, well, that son’s gone. This is a different one. And then you have to live without that, like hmm. I don’t know anything about this. I have nothing, I have to learn what this means. I don’t know.”

The uncertainty that resulted from their child’s unknown future also affected some mothers’ bond or social relationship with their child. For example, the news of her child’s DS diagnosis suddenly altered Mia’s belief in her ability to parent him: “I was really shocked. I was really upset… [I] kind of felt like, ‘Well, now what do I do with him?’ Suddenly, I didn’t know how to take care of him.” This initial reaction was based on uncertainty about their child’s needs and what it would mean to be the mother of a child with DS.

In addition to the social implications of their child’s DS diagnosis, mothers communicated uncertainty regarding many facets of managing unknown health concerns that might impact their child’s physical quality of life, including physical abilities and comorbid conditions, their child’s ability to care for themselves, and their child’s duration and quality of life. Mothers also reflected on whether their child might be able to attend school, and if so, the services that might be required. Fiona summarized many of these health-related unknowns:

“There’s so many question marks. Is she going to be healthy? Is she going to need a feeding tube? Is she going to need heart surgery? Is there going to be a dual diagnosis, which complicates things?”

Fiona’s quote highlights an overall concern for her child’s physical health and the long-term consequences that might accompany those potential health issues. Samantha also describes concerns surrounding uncertainties with physical quality of life:

“How disabled was she going to be? What were we going to go through?… I stayed up at night one night and read all those things [that] can happen to a child with DS, and I remember crying all night. And so that night I just am sitting there and imagining the worst, ‘What if she gets leukemia? What if she gets a thyroid thing?’”

Taken together, these mothers’ narratives highlight the ways in which societal and personal stereotypes led to uncertainty regarding a child’s social acceptance, as well as how managing health concerns with uncertain outcomes might impact a child’s physical quality of life in the future, both of which exacerbated the complicated process of navigating a DS diagnosis.

#### Anticipating family-centered challenges and adjusting familial expectations

Mothers described uncertainty regarding how having a child with DS might impact their family life. Though each family experienced unique circumstances, and thus consistent subthemes were not identified, many mothers experienced generalized uncertainty about how family life might differ after learning of their child’s DS diagnosis. For example, Cindy reflected: “I don’t know anybody in my family who has this… We haven’t dealt with anything like this before. No one knows what to expect.” Mothers also expressed uncertainty resulting from their attempts to anticipate how their child’s DS diagnosis might alter family life. Connie recalled:

“A billion things run through your mind, right?… I’ve worked so hard to get to where I am, will I be able to continue doing that? What does this mean for our future? Will we be able to travel? Will we be financially strapped? What does this mean? We had literally no idea what it meant.”

Connie describes feeling like she had a plan for her family’s future that was replaced by a series of questions and unknowns after learning of her child’s DS diagnosis. Some mothers felt that this uncertainty resulted from a lack of knowledge about DS or misperceptions about what the diagnosis entailed. For example, Amy explained: “I had all of these incorrect thoughts… like he’s going to be homebound [his] entire life. Our whole life stops now.”

Uncertainty also resulted from considering the impact of the child’s DS diagnosis on particular family members. Mothers expressed concern about the impact of the diagnosis on their other children, with some citing concerns that older siblings in particular might reject the child with DS. As Riley recalls: “I was terrified that my teenagers would… shun having a sibling with special needs.” Though Riley later shared that her fears were ultimately unfounded, this uncertainty persisted throughout her pregnancy. Mothers also described the uncertainty experienced by their partners when it came to welcoming a child with special needs into their family. For example, Nadine stated:

“He hadn’t really talked about it much, but I could see he was constantly worried. But I think to him it was more worried about the health part of the baby and whether he would be able to take care of him and help and really make sure that this baby had everything that he would need.”

As these examples demonstrate, mothers experienced increased uncertainty as they attempted to anticipate and meet the needs of other family members at the same time as their own.

In sum, the four themes that capture mothers’ primary sources of uncertainty in our dataset reflect the myriad ways that these mothers’ lives–already in flux with the addition of a newborn to the family–were unexpectedly changed as they learned of their child’s DS diagnosis and adapted to their birth. Yet, even as they faced uncertainty resulting from navigating dissonance during diagnosis, managing disclosure of the diagnosis to others, anticipating child-centered challenges and adjusting developmental expectations, and anticipating family-centered challenges and adjusting familial expectations, mothers used multiple tools to manage the uncertainty they experienced.

### Management tools

Management tools coalesced into four overarching themes seen in mothers’ narratives: finding balance between parents and providers during diagnosis, reducing knowledge gaps by seeking information, pursuing support and building positive interpersonal relationships, and pursuing support and building positive relationships in DS communities. As before, subthemes within two of these themes (establishing collaboration between parents and providers during diagnosis and seeking support and building positive relationships in familial and friendship experiences) are shown in bold text the first time they are introduced.

#### Finding balance between parents and providers during diagnosis

Although navigating dissonance during diagnosis served as a source of uncertainty for many mothers, this was also a context in which mothers attempted to manage their uncertainty as they jointly engaged with, relied on, and sometimes sought out particular medical professionals. Two subthemes emerged that reflected provider-centric interactions and behaviors: a **match in communication and knowledge between providers and parents**, and providers who presented **a balanced perspective on DS**. An additional subtheme was parent-centered: **realization of maternal agency**.

The provision of clear, factual communication about DS during the delivery of the diagnosis was helpful for mothers, as seen in this exchange between Rita and her doctor: “She says, ‘The baby tested positive for Trisomy 21.’ I’m like, ‘That’s DS, right?’ And she says, ‘Yes,’ and I was like, ‘OK.’” In this circumstance, the match between the level of medical information provided and Rita’s background knowledge helped to reduce her uncertainty, as did efforts by medical professionals to clarify and further explain the diagnosis. For example, a social worker provided additional clarification to Susan after she learned of the official diagnosis from a geneticist:

“[She] really made things more at ease and more comfortable. She actually explained some things, which was appreciated because everyone else just kind of gave us a diagnosis and then went on… It was a really nice, nice thing to have.”

Interactions with medical providers that were supportive and featured a balanced perspective incorporating both potential challenges as well as positive outcomes associated with DS also served to resolve uncertainty for these mothers. In contrast to Clara’s experience with a medical provider who focused on stereotypes and the negatives associated with a DS diagnosis (p. 12), Connie’s doctor provided a more balanced assessment:

“‘Having a diagnosis of DS doesn’t mean anything. It just means that he’ll do things more slowly. But I know plenty of adults with DS who turn out fine.’ So… he presented it as this very positive thing.”

A balanced diagnosis was generally well-received by mothers and allowed for better management of maternal uncertainty. It is also important to highlight that mothers specifically reflected on nurses as professionals who communicated support, particularly emotional support, throughout their birth and diagnosis experience. Mothers appreciated that some nurses went above and beyond their professional role to offer reassurance and guidance about the potential positive impacts of a DS diagnosis, as seen in Amy’s story:

“The nurse that had admitted us into the OB department actually had a son with DS, who was, I think, five at the time. So she came in and talked [to] us pretty much immediately after all of that and just let us know it’s not the end of the world. It’s unexpected, but it’s not anything horrible. And so I thought that was just super helpful. And I’m so glad that she was there and that just kind of worked out that she was our nurse.”

Other exchanges with nurses were also instrumental in terms of support offered, as Nadine says of her nurses’ willingness “to come and help me and teach me this again” anytime, day or night, during the hospitalization following her son’s birth. Provision of emotional and instrumental support from some healthcare professionals appeared to balance out more negative encounters with others, ultimately helping mothers process their child’s diagnosis and adapt to their birth.

Maternal agency also emerged as a tool for managing uncertainty as mothers drew upon their experiences and identities to take the lead and proactively manage medical situations. Mothers capitalized on their occupational identities, adopted an identity as an advocate, and called upon internal strength and resources. For example, some mothers accessed specialized medical services for their child as a result of their proactive communication with healthcare providers, as indicated by Samantha:

“The good news of finding out prenatally was that we were able to prepare, so rather than get whoever was the yahoo that was on call, we found the best surgeon in the state and scheduled the surgery with him and went to the hospital where he had privileges.”

Other mothers actively invoked occupational and life experiences that allowed them to better manage their uncertainty surrounding their child’s diagnosis. In these ways, mothers called upon their previous life experiences and internal resources to increase their agency and manage their uncertainty surrounding their child’s DS diagnosis.

#### Reducing knowledge gaps by seeking information

Mothers sought out information to learn more about their child’s diagnosis and potential development from books, articles, and the internet. After learning of their child’s diagnosis, mothers indicated that they wanted to “prepare,” “learn more,” and “research stuff,” or referred to “reading about everything,” with Janice explaining: “We got [to] reading all about DS… me and my husband took a crash course, what DS is, what do we need to do.” Because of the wide array of information-seeking behaviors and media use to find information, no specific subthemes were identified.

Mothers discussed seeking information from online resources both alone or with their partner. Due to the ease of accessing online content, some mothers even began searching for more information while still in their doctor’s office (after a prenatal diagnosis) or in the hospital following childbirth (in the event of a postnatal diagnosis). Although not true for all of the mothers in our sample, some mothers mentioned that seeking information answered questions and reduced uncertainty in a positive way. Nadine said:

“I started reading actual websites that are more positive and that actually give you real information instead of scared things…. We felt better, both of us.”

As Nadine discussed, seeking information and finding answers to her questions not only reduced her uncertainty but also allowed her a glimpse into the positives associated with having a child with DS. However, for other mothers, seeking information from online sources seemingly reduced their uncertainty while simultaneously increasing their anxiety. For instance, Fiona explained:

“We were Googling a lot…. My baby’s in the NICU, I’m in the hospital room by myself…. You’re looking online, what’s this going to be like, what’s going to happen, who is she going to be. And there’s not, I mean, there’s some positive stuff, but I feel like it was mostly negative, kind of scary things to read about.”

As indicated, mothers varied with regard to the sources from which they received information, their own unique interpretation of and ability to cope with the information they learned, and their comfort level with the inherent unknowns that accompanied a DS diagnosis.

#### Pursuing support and building positive interpersonal relationships

Mothers engaged in conversations with others to manage some of the uncertainty resulting from the birth and diagnosis of their child with DS. Clear themes in the data revealed mothers relied on and appreciated this communication both **within the family** and **across broader social networks**.

Mothers discussed reaching out to talk with their family, including their romantic partner, particularly if they were alone when they received their child’s diagnosis. Mothers also described the ways in which their partner’s support allowed them to process their child’s DS diagnosis and subsequent challenges. As Nadia described:

“I’ll always remember the words my husband said to me.… We can’t change the world, but we can… help her be as prepared for the world as she can be. And I think those words were really kind of what helped me change my outlook.”

Mothers’ families of origin (e.g., parents, siblings, aunts, uncles) also provided reassurance that helped these women manage their uncertainty about their child’s diagnosis. For example, Maria’s sister initially reacted to the news by saying: “‘That’s OK. He’s going to be OK and you’re going to be OK.’” Similarly, Nadine noted of her family: “They were just there for me, even if they weren’t here, here, but I was talking to them a million times a day.”

Mothers expressed internalizing the messages from those conversations, which allowed them to reframe the situation. This process is evident in Riley’s experience, as she was told during her pregnancy that her child might have a different genetic condition that is commonly fatal. A conversation with her mother helped her manage her uncertainty about her child’s precarious situation:

“But my mom finally came back to me and she told me, she said, ‘Riley, he’s so perfect. I think he’s fine.’ She said, ‘He does look like he might have DS, but otherwise he looks very healthy. Like he’s crying, he has color, you know he’s really cute.’ So that calmed me down a little bit, just knowing, ‘OK, he’s crying and screaming.’”

Riley’s mother emphasized the signs indicating that her newborn son was healthy, helping Riley reframe her perspective on a DS diagnosis. Riley goes on to describe how juxtaposing her son’s DS diagnosis with the potentially fatal genetic condition they were expecting helped her and her husband reframe their perspective:

“We had already dealt and processed that we might not even bring him home. So having DS was like, ‘Oh, well, he can live with this. I won’t have to bury my child.’ I mean, it’s not the ultimate what you want, but I think we had a different mindset with it. Instead of being crushed, we kind of grieved my whole pregnancy, so that by the time he was born, it was almost a relief that that’s all it was.”

In addition to communication within the family, mothers discussed how conversations across broader social support networks were central to reducing their uncertainty. Friends provided support to mothers as they processed their child’s diagnosis and as they considered whether their child would be loved and accepted by others. As Nadine recalled: “Other friends came to see him. Everybody was just, ‘God, he’s so beautiful.’ It’s just so much love for all of us. For him.”

For some families, it was helpful to share their child’s diagnosis more openly, perhaps to alleviate the stress associated with having to repeatedly share the diagnosis during individual interactions. Sharing the information in this way may have also allowed others time to process the child’s DS diagnosis, which, as Ramona describes, may have allowed them to better support and celebrate important life events, such as the child’s birth: “We told people while I was still pregnant, so everybody kind of had time to digest the information, and once she was born… she was just the baby.” Similarly, Dora engaged in conversations across her immediate support systems and posted to social media to inform her entire social network of her child’s diagnosis:

“I told everybody the same day that I found out. Well, I told my husband. After I told him, I told his parents, my parents, and I also told Facebook. I just felt like I wanted to get it out and just tell everybody. So, they found out that same day too.”

In sum, communication within the family provided mothers with a familiar space to explore and make sense of their child’s DS diagnosis. The messages and support they received from their family were internalized and allowed for reframing that ultimately helped reduce their uncertainty. Communication across broader social networks allowed mothers to inform many people of their child’s diagnosis at once and provided an additional source from which they could receive positive messages and support.

#### Pursuing support and building positive relationships in DS communities

Mothers’ ability to manage their uncertainty was enhanced by the support they received from the DS community in particular. Some of the support provided by members of the DS community was found online within intentionally-formed birth groups (i.e., groups of mothers who had children with DS born around the same time). Melanie reflected: “So there [were] other mothers, all mothers of babies with DS, and right around my age, and they were amazing. They were really wonderful.”

Other mothers stated that connecting in-person with other parents was “the biggest help” in navigating various aspects of uncertainty. Tammy stated: “Getting to see the other kids, and just interacting with them has helped us so much.” Melanie and Tammy both found solace in receiving support from other mothers of children with DS who were experiencing or had previously experienced similar situations, and, as such, could provide encouragement and advice needed to navigate the nuances of raising a child with DS.

Within some families, different aspects of community support were critical for each parent to manage their own uncertainty. For example, after receiving a prenatal diagnosis of DS, Emma and her partner considered whether they should terminate the pregnancy. Whereas the mother’s uncertainty was resolved through involvement with a DS association and interactions with other parents, her husband remained uncertain. For him, the critical factor was being able to meet individuals with DS during a local event:

“That’s when we actually saw children with DS and parents and got to talk to them… I think this was the turning point for my husband… when he saw those other parents and their children.”

These quotes highlight the importance of integration into and support from the DS community. Other mothers of children with DS, as well as involvement in DS organizations and associations, both online and in-person, were imperative in providing the necessary support for negotiating the uncertainty of a DS diagnosis.

### Variability based on diagnosis timing

Fifty-nine percent of mothers received a postnatal diagnosis of DS for their child whereas 41% received a prenatal diagnosis.Therefore, we explored whether there was variability in the themes of uncertainty and uncertainty management tools in relation to when the diagnosis was provided.These descriptive data are shown in Table 2, and we return to potential implications of, and future directions for, comparisons such as these in the Discussion.

**Table 2:** Sources of uncertainty and management tools by timing of diagnosis.

	Group	
	Prenatal (n = 18)	Postnatal (n = 26)
Sources of uncertainty		
Navigating dissonance between parents and providers during diagnosis		
Mismatch in communication or knowledge between providers and parents	44%	38%
Provider focus on the negative aspects of DS	17%	12%
Managing disclosure of the diagnosis to others	11%	15%
Anticipating child-centered challenges and adjusting developmental expectations		
Navigating societal and personal stereotypes	33%	38%
Managing health concerns	33%	31%
Anticipating family-centered challenges and adjusting familial expectations	22%	31%
Management tools		
Finding balance between parents and providers during diagnosis		
Match in communication and knowledge between providers and parents	39%	42%
Providing a balanced perspective on DS	22%	27%
Realization of maternal agency	33%	8%
Reducing knowledge gaps by seeking information	61%	35%
Pursuing support and building positive interpersonal relationships		
Within the family	33%	50%
Across broader social networks	22%	8%
Pursuing support and building positive relationships in DS communities	50%	31%

## Discussion

The present study features a novel thematic analysis of mothers’ narratives regarding the birth and diagnosis of their child with DS that contributes to the literature by identifying four sources of uncertainty and four ways in which mothers communicated that they managed their uncertainty. Our findings revealed that some aspects of these mothers’ experiences served as both a source of uncertainty and as a management tool (e.g., dissonance or balance in the context of the diagnosis delivery and communicating about the child’s diagnosis with family and friends), though the long-term child-centered and family-centered unknowns associated with a DS diagnosis were consistent sources of uncertainty. Finally, exploratory data that described themes of uncertainty and uncertainty management tools for mothers with a prenatal or postnatal diagnosis for their child provide an intriguing path for future research to further explore whether targeted efforts for each of these groups could be made to provide them with the tools they need to best manage their child’s DS diagnosis immediately after the diagnosis is provided and over the long term.

### The context during diagnosis: Dissonance as a source of uncertainty, and balance as a management tool

Some interactions with medical professionals acted as sources of uncertainty whereas others facilitated mothers’ ability to manage the uncertainty surrounding their child’s birth and DS diagnosis. Although there have been consistent recommendations for medical professionals to more sensitively communicate the details of DS diagnoses [6, 7], mothers in this sample recounted how some medical providers contributed to their uncertainty, either due to dissonance caused by an abrupt or poorly-timed delivery of the diagnosis or by focusing on the negative aspects of DS. In contrast, mothers expressed being able to manage their uncertainty better when a balance was found with their provider as they were given a clear, understandable diagnosis provided in a supportive context. These findings underscore previous calls for sensitivity and intentionality in diagnosis delivery. Mothers expressed appreciation when medical providers offered a realistic, yet balanced, perspective of DS that did not focus only on negatives. Though a balanced view can be difficult to provide given variability in health and social outcomes for individuals with DS, it is important to discuss the range of possibilities with families [29]. Mothers also managed their uncertainty better when they were empowered to collaborate with medical professionals and used their previous life experiences to advocate for themselves and their child. Although several studies have examined under what conditions adult patients ask questions of their medical providers and how to encourage that behavior [30, 31], future research should examine the specific language that medical providers can use to facilitate communication and encourage families to ask questions and exercise agency regarding their child’s DS diagnosis.

In our sample, although physicians provided emotional support to families at times, mothers more often recognized their nurses as filling this role. The extra time and effort that goes into many patient-medical professional interactions has been documented and indicates that compassion, empathy, and patients being “seen” as a person contribute positively to these relationships [32, 33]. We are mindful that some of these personal, positive interactions are beyond the requirements expected of medical professionals, and that parent-nurse partnerships may be limited by structural barriers such as hospital policies [34]. In our sample, however, nurses were particularly impactful in helping mothers manage the uncertainty experienced after receiving a DS diagnosis for their child and we wish to acknowledge their positive influence.

### Social interactions as a source of uncertainty and management tool

As mothers processed the birth and diagnosis of their child with DS, they communicated with their partner, families, friends, broader social networks, and the DS community. Mothers expressed uncertainty about sharing the news of their child’s birth and managing the disclosure of their diagnosis, in part because they were unsure of others’ reactions. Past research has shown that individuals hold multifaceted views of DS and those with the condition, and those views are associated with individual beliefs about prenatal testing as well as pregnancy continuation or termination [35]. In our sample, some mothers had to contend with other people’s negative responses to the news of their child’s DS diagnosis; the responsibility to counsel others compounds an already challenging situation for parents [9]. However, mothers’ conversations with individuals across various relationships and within the DS community also served to manage their uncertainty. Mothers primarily turned to their partners for support but also actively engaged with their parents, adult siblings, and extended family to help reduce uncertainty.

These mothers’ narratives also demonstrate the importance of DS organizations as providers of basic information about DS and as a mechanism through which families connect with one another. Mothers were able to build relationships and share their experiences and hopes for the future with parents in similar situations, and children were able to build relationships with each other. Furthermore, these organizations helped parents acquire the communication skills and tools needed to advocate for their child across multiple contexts.

### Reducing knowledge gaps by seeking information may decrease or increase uncertainty

When mothers felt they lacked an adequate amount of knowledge about the DS diagnosis, many reported seeking additional information, most commonly online. Although individuals seek information with hopes of reducing uncertainty, finding new information sometimes results in an increase in uncertainty due to the negative nature of the information obtained. In some cases, mothers may receive conflicting information about what the DS diagnosis entails, which can then produce additional uncertainty. Mothers may also become overwhelmed by the negative portrayal of DS in society at large, leading to increased anxiety about long-term possibilities for their child’s future. At the same time, past research has shown that receiving potentially conflicting and complicated information in a healthcare setting often allows patients to become personal advocates who are highly knowledgeable about their condition [36]. In this way, what begins as a large source of uncertainty may end up becoming a platform for advocacy and education, as we see in many of these mothers’ stories. Future work should address whether initial uncertainty and information-seeking to increase knowledge was utilized as a tool for self-empowerment, ultimately impacting how these mothers came to manage their uncertainty [17].

### The future as a source of uncertainty

Mothers described how the unknowns associated with a DS diagnosis–for their child and their family, in the present and in the future–were a significant source of uncertainty. Although mothers attempted to manage their uncertainty by seeking out information, the infinite number of unknowns regarding what the diagnosis meant for their child and for their family could not be fully answered or managed. Building on work that has described parents’ sources of grief and loss resulting from a DS diagnosis [8, 37], our data suggest that uncertainty is amplified because mothers cannot know the long-term impact of a DS diagnosis on the child’s life, on specific family members (such as siblings), and on the family unit as a whole. Future research is needed to identify and implement specific targeted approaches that may help families better manage the uncertainty associated with the impact of the diagnosis on siblings and the broader family unit as well as their child’s long-term social and physical well-being.

### Practical implications

We emphasize that the sources of uncertainty these mothers experienced did not always operate independently; rather, they often co-occurred and likely compounded stress. For example, the manner in which the diagnosis was delivered may have contributed to mothers’ feelings of uncertainty when sharing the news with others, which may have also been compounded by the unknown long-term implications for their child’s future. Some mothers were also seemingly so overwhelmed with their new reality that they did not know what questions to ask, how to ask them, or how to care for their child. These sources of uncertainty seemed to culminate in uncertainty about the impact of this diagnosis on the family–as a whole and for specific family members.

Thus, we echo previous calls to providers, encouraging them to be mindful of the setting, content, and tone of their communication with families when delivering a DS diagnosis and in subsequent conversations. With expanded prenatal testing, diagnosis delivery may fall more frequently to obstetricians and other healthcare providers who do not provide care for children with DS and may be unprepared to answer specific questions about this condition. Providers must be aware that they are not just delivering a one-time diagnosis to parents; rather, parents will forever remember and appreciate (or spurn) the role that providers played at a critical time in their family’s history. Providers must also be aware that parents seek information from multiple sources and that some of the information received may be inaccurate or out of date. Providers should be prepared to direct parents’ online searches to appropriate sources, to counter false or misrepresented depictions of DS, and to sensitively explain to parents that no one knows their child’s future and no source will fully reduce their uncertainty. Families often sought out local and online DS associations on their own, but having this information readily available for families is critical as these organizations provide invaluable skills and information to parents.

Future work should continue examining structural barriers that may prohibit mothers from receiving the care they or their children need. These barriers may include limited access to medical testing (because mothers do not qualify or because insurance will not cover it) and limitations on which outside resources are provided to parents at the time the diagnosis is provided. Although less common in this sample, other populations (e.g., those of lower socioeconomic status, those without standard health insurance) may encounter more significant structural barriers that represent additional sources of uncertainty and contribute to increased stress. Access to specialized healthcare services is privileged (e.g., reference [38]), which is a larger systemic consequence of an inequitable healthcare system [39]. As such, it would be advantageous to families to remove these barriers as much as possible during what is an already challenging time.

Future researchers should also explore whether children’s physical health issues and the presence of comorbid conditions are associated with maternal uncertainty. One possibility is that the presence of significant co-occurring conditions or comorbidities might result in more uncertainty for these parents, but it is also possible that parents recruit additional management tools in such scenarios. We were limited in our ability to address this question, as our inclusion criteria required mothers to have a child between the ages of 1–17 years old at the time of the study. This selection bias did not allow us to examine sources of uncertainty or management tools in families whose child had passed away or in families whose child was born in a different generation and thus may have had very different birth and diagnosis experiences.

Finally, though exploratory, our data suggest that mothers who received a postnatal diagnosis of DS for their child may be less prepared to manage their uncertainty relative to mothers who receive a prenatal diagnosis. Although mothers who received a prenatal diagnosis frequently reported uncertainty arising from the manner in which the news was delivered, receiving the diagnosis during pregnancy may have allowed them more time to manage uncertainty by seeking out information to reduce knowledge gaps, enacting agency to access the care they needed, and receiving support from their wider social networks and the DS community. The implementation of these management tools may have afforded mothers the opportunity to establish substantial in-person medical, emotional, and physical support even before their child’s birth. However, mothers who received a postnatal diagnosis relied heavily on personal connections within existing family networks, and perhaps sensing these mothers’ needs, providers offered supportive information and stepped outside their roles to help them cope.

Together, our findings suggest that targeted efforts should be made to understand the unique sources of uncertainty that families of children with DS may experience and to assist families in identifying and accessing the specific tools they need to best manage their child’s DS diagnosis. Future intervention efforts should also account for differences in the experience of uncertainty and the ways in which it is managed in relation to when a DS diagnosis is made, ultimately allowing all families to joyfully welcome their new baby while most successfully managing the uncertainty they encounter as they navigate their changing life circumstances.
